# Changes of Functional and Effective Connectivity in Smoking Replenishment on Deprived Heavy Smokers: A Resting-State fMRI Study

**DOI:** 10.1371/journal.pone.0059331

**Published:** 2013-03-19

**Authors:** Xiaoyu Ding, Seong-Whan Lee

**Affiliations:** 1 Department of Computer Science and Engineering, Korea University, Anam-dong, Seongbuk-ku, Seoul, Republic of Korea; 2 Department of Brain and Cognitive Engineering, Korea University, Anam-dong, Seongbuk-ku, Seoul, Republic of Korea; Aalto University, School of Science, Finland

## Abstract

Previous researches have explored the changes of functional connectivity caused by smoking with the aid of fMRI. This study considers not only functional connectivity but also effective connectivity regarding both brain networks and brain regions by using a novel analysis framework that combines independent component analysis (ICA) and Granger causality analysis (GCA). We conducted a resting-state fMRI experiment in which twenty-one heavy smokers were scanned in two sessions of different conditions: smoking abstinence followed by smoking satiety. In our framework, group ICA was firstly adopted to obtain the spatial patterns of the default-mode network (DMN), executive-control network (ECN), and salience network (SN). Their associated time courses were analyzed using GCA, showing that the effective connectivity from SN to DMN was reduced and that from ECN/DMN to SN was enhanced after smoking replenishment. A paired *t*-test on ICA spatial patterns revealed functional connectivity variation in regions such as the insula, parahippocampus, precuneus, anterior cingulate cortex, supplementary motor area, and ventromedial/dorsolateral prefrontal cortex. These regions were later selected as the regions of interest (ROIs), and their effective connectivity was investigated subsequently using GCA. In smoking abstinence, the insula showed the increased effective connectivity with the other ROIs; while in smoking satiety, the parahippocampus had the enhanced inter-area effective connectivity. These results demonstrate our hypothesis that for deprived heavy smokers, smoking replenishment takes effect on both functional and effective connectivity. Moreover, our analysis framework could be applied in a range of neuroscience studies.

## Introduction

Drug addiction is a complex brain disorder characterized by the compulsive consumption of a deleterious drug, the fundamental mechanisms of which are now studied in the neuroscience field. With the aid of fMRI, researchers have explored the underlying impact on brain functions caused by multiple addiction drugs including opium [1], cocaine [2]–[3], heroin [4]–[5] and more. Among these, smoking addiction is also commonly researched due to the wide range of tobacco users and the public sale of cigarettes. fMRI experiments within the study of drug addiction can be typically categorized into two types according to their experimental design: task-related experiments [6], and the so-called “resting-state” experiments [7].

In a task-related experiment, fMRI data are acquired while participants are performing a certain task. A widely used task in smoking research is the visual cue-exposure task, in which smoking-related visual cues and neutral cues are presented to participants during scanning [8]–[10]. Although this type of study is essential for delineating the acute effects of smoking on circumscribed brain areas, the existence of intrinsic and spontaneous hemodynamic activity in resting-state may reveal the inherent mechanisms of smoking on brain functions more efficaciously. Compared to the task-related design, the resting-state experiment design has a prominent advantage in that the data collection is relatively quick and straight-forward. The participants lie passively without any cognitive or behavioral tasks during scanning. Moreover, identified resting-state neural mechanism features are less likely to be confounded by subtle differences in specific task designs. For these reasons, a resting-state experiment design has been widely adopted in smoking studies [11]–[13].

Two types of dissociable large-scale brain networks have been explored in terms of the effect caused by smoking in both task-related and resting-state conditions [14]–[17]. One is the “default-mode network” (DMN), which is a “task-negative” network (TNN) that shows activity increases during resting-state condition. This contains two parts: the anterior DMN (aDMN), e.g., within the medial/superior frontal areas and anterior cingulate cortex (ACC), and the posterior DMN (pDMN), e.g., within the posterior cingulate cortex (PCC) along with the precuneus and inferior parietal lobe [18]. The other type is termed the “task-positive” network (TPN), which exhibits increased activity in performing tasks. In the absence of explicit task demands, two distinct TPNs appear to exist: one is the “salience network” (SN) within the anterior insula and ACC, the other is the “executive control network” (ECN) which comprises the lateral prefrontal and parietal regions [19].

These brain networks appear in such a way that they contain some remote neuronal circuitries which show high temporal correlations across each other. In neuroscience studies, this kind of temporal correlation between spatially remote neurophysiological events is defined as functional connectivity [20]. To examine functional connectivity using resting-state fMRI data, one solution is the “seed voxel” approach [21]: a voxel together with its surrounding small region of interest (ROI) is chosen as the seed, whose average time-series then serves as a reference for calculating the temporal correlation with the signal in each voxel. An inherent limitation of this method is that the obtained activity map depends highly on the selection of the seed voxel. In order to obviate the interference caused by the selection of seed voxel, independent component analysis (ICA) [22], which is a blind source separation method under the assumption that the extracted components are statistically independent, has been introduced and widely applied to analyze functional connectivity in resting-state fMRI data [23]–[24]. The ICA method results in spatially independent patterns together with their corresponding time courses.

Corresponding to functional connectivity, another ensemble called effective connectivity is defined as the causal influence from one neuronal system to another [25]. Granger causality analysis (GCA), which is equivalent to a correlation-based method [26], has been proposed for estimating the causal interactions of information flows. Previous fMRI studies used the GCA approach to explore the interactions among brain networks [27]–[28] or brain regions [29]–[30] identified by ICA during task-related or resting-state fMRI experiments.

To the best of our knowledge, no previous study has explored the effect of smoking on effective connectivity. This study therefore aims to investigate the influence of smoking on both functional and effective connectivity, and it is exploratory in nature. We hypothesized that the effect of smoking replenishment on deprived heavy smokers would influence both functional and effective connectivity. Considering effective connectivity, we further hypothesized that the causal interactions among TNNs (i.e., aDMN and pDMN) and TPNs (i.e., ECN and SN), as well as those among the crucial brain regions as revealed by functional connectivity analysis, would be changed significantly. For this purpose, we conducted a resting-state fMRI experiment with conditions of smoking abstinence followed by conditions of smoking satiety on heavy smokers. We propose an analysis framework combining ICA and GCA so as to give attention to both functional connectivity and effective connectivity, including brain networks and crucial brain regions. In our framework, we first investigate the functional connectivity of TNNs and TPNs using group ICA followed by a paired *t*-test on the ICA resulted spatial patterns. The corresponding time courses are used as inputs to GCA to reveal the causal interactions among brain networks. Several ROIs are selected based on the previous paired *t*-test results, and their causal relationships are also explored using GCA.

The rest of this paper is organized as follows: in the following section we describe the resting-state fMRI experiment and our analysis framework in detail. We then present the experimental results together with related analysis. We later discuss several interesting aspects of our findings and form a conclusion.

## Materials and Methods

### Ethics Statement

This study was approved by the Institutional Review Board of Korea University. All participants provided written informed consent and received monetary compensation for their participation.

### Participants

Twenty-one right-handed male smokers (mean age = 25.86 years ±2.10, range = 22∼30 years) were enrolled as participants in this study. All participants were heavy smokers (>5 years, >15 cigarettes/day). They were firstly interviewed to verify they had no history of neurological or psychiatric illness, and were also examined to confirm no recent alcohol consumption or other recreational drug use. All participants completed the Fagerström Test for Nicotine Dependence (FTND) [31] during initial screening (mean score = 6.19±0.93, range = 5∼8). Each participant was deemed free of structural abnormalities following review of anatomical MRI scans.

### Procedure

All participants were scanned for two resting-state sessions [32]. In the first session, the participants had abstained from smoking for more than twelve hours, and the levels of exhaled carbon monoxide (CO) were measured using Bedfont smokerlyzer piCO^+^ (mean = 9.62 ppm ±3.53, range = 4∼15). They took the resting-state scanning with eyes closed. After the first session, they were allowed to smoke until they felt satisfied. The levels of exhaled CO were measured again (mean = 22.86 ppm ±8.05, range = 11∼42), which showed a distinct increase for each participant compared to that measured in the first session (see Table 1). They then took the second session resting-state scanning still with their eyes closed. In the remaining part of this paper, the first and second session will be denoted as “RS_abs” and “RS_sat” respectively.

**Table 1:** The FTND score, as well as the CO-levels tested in RS_abs and RS_sat for each subject.

Subject ID	FTND	CO (RS_abs)	CO (RS_sat)
001	5	14	28
002	6	15	42
003	8	11	24
004	6	13	26
005	6	12	25
006	6	11	26
007	6	7	21
008	5	8	17
009	7	8	21
010	6	6	15
011	6	11	21
012	8	15	42
013	8	5	26
014	6	4	11
015	6	13	23
016	7	12	26
017	6	7	19
018	6	6	13
019	5	5	13
020	5	7	17
021	6	12	24

### Data Acquisition and Preprocessing

Blood oxygenation level-dependent (BOLD) contrast, single-shot, T_2_^*^-weighted, gradient-echo planar imaging (EPI) data were acquired in an interleaved multi-slice mode using a 3T clinical scanner (MAGNETOM Trio Tim, Siemens) with a 12-channel head coil. Each scan session contained 155 volumes of the whole brain in 36 axial slice acquisition (TR = 2000 ms, TE = 30 ms, flip angle = 90°, field of view = 240×240 mm^2^, voxel size = 3.8×3.8×4.0 mm^3^). The first 5 volumes were discarded from further analysis to allow for magnetic equilibration. Additional high-resolution T_1_-weighted anatomical MRI scans (MPRAGE) were acquired with a 32-channel head coil for each participant (TR = 1900 ms, TE = 2.32 ms, flip angle = 9°, field of view = 240×240 mm^2^, voxel size = 0.9×0.9×0.9 mm^3^, 192 slices).

Raw EPI data were preprocessed using the SPM5 toolbox (http://www.fil.ion.ucl.ac.uk/spm). Slice timing differences were corrected for each volume. The functional volumes were realigned to the first volume for head movement correction, and then normalized into standard MNI (Montreal Neurological Institute) space with 3×3×3 mm^3^ voxels. After linear trends were removed, these data were band-pass filtered (0.01–0.08 Hz) to reduce the effect of low-frequency drift and high-frequency physiological noise [30]. Finally, they were smoothed with a 8×8×8 mm^3^ full width at half-maximum (FWHM) Gaussian kernel.

### Overview of Analysis Framework

**Figure 1:**
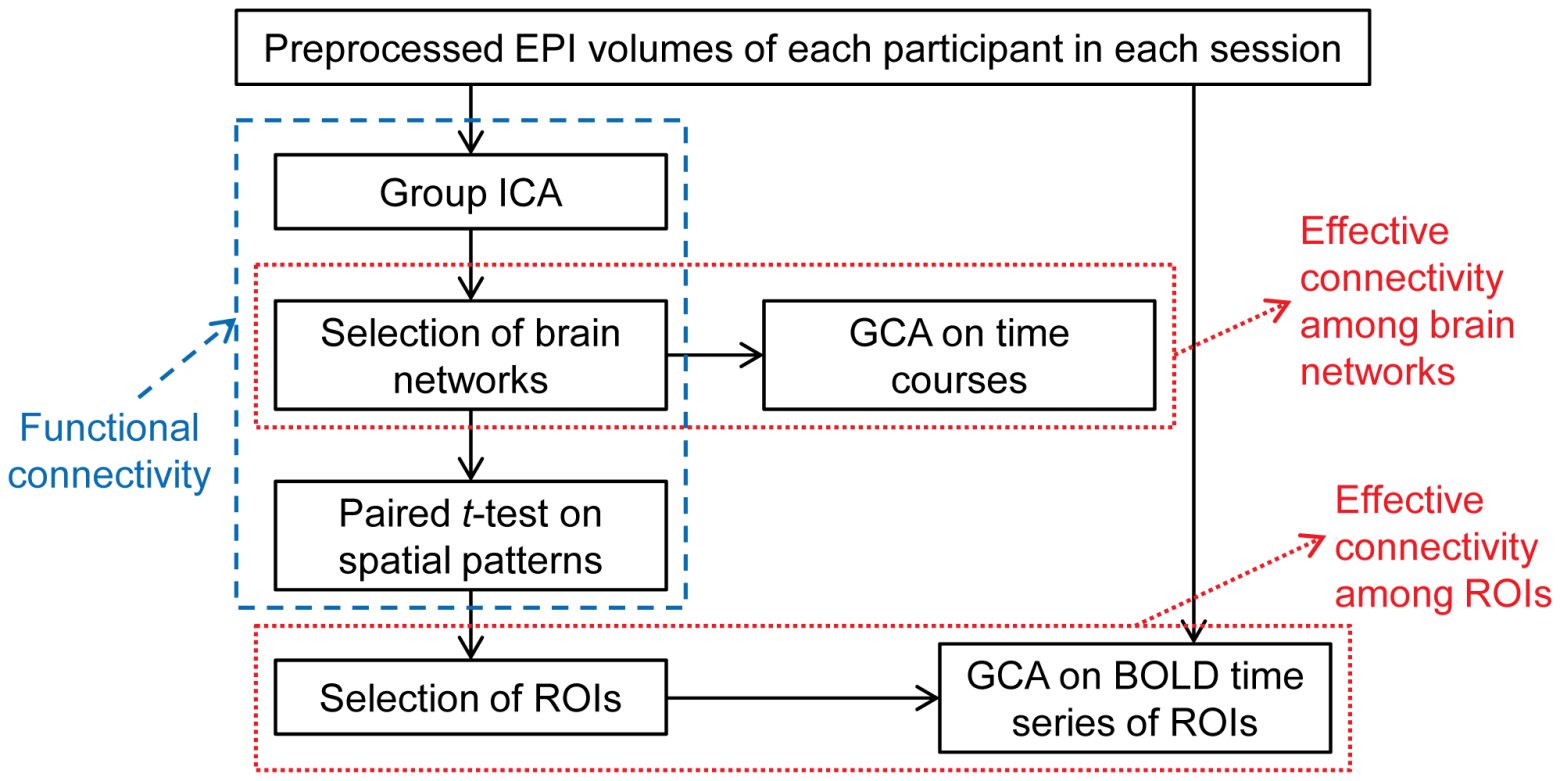
An overview of the proposed analysis framework.

An overview of the proposed analysis framework is illustrated in Fig. 1. After preprocessing the raw EPI data, a group ICA method was applied on the temporally concatenated dataset of each participant in each session. Decomposed brain networks of TNNs (i.e., aDMN and pDMN) and TPNs (i.e., ECN and SN) were selected for further analysis. Their spatial patterns were examined using a paired t-test to investigate the changes of functional connectivity caused by smoking replenishment. The corresponding time courses were used as inputs to GCA to reveal the causal interactions among the selected brain networks. Several ROIs were selected based on the result of previous paired *t*-tests, and their BOLD time series were analyzed using GCA to explore the effect of smoking on causal interactions among critical brain regions. The following parts will describe this framework in detail part by part.

### Independent Component Analysis (ICA)

We adopted a group ICA [33] method to execute the ICA approach. Group ICA is commonly used for making group inferences from fMRI data of multiple subjects. In our study, this was carried out using the GIFT toolbox (http://icatb.sourceforge.net) for not only estimating individual spatial patterns but also facilitating investigation of group differences under the same study condition. The individual datasets were temporally concatenated (datasets of RS_abs followed by datasets of RS_sat) and reduced for computational feasibility through three stages of principal component analysis to obtain a dimension reduced final dataset, which was then decomposed by ICA (Infomax algorithm [34]) into twenty-three spatially independent components. Here, the number of components was estimated using the minimum description length criteria [35], and also accordant with the common settings in previous ICA-GCA researches [27]–[30]. The Infomax algorithm was repeated twenty times with randomly initialized decomposition matrices and the same convergence threshold using ICASSO approach [36] in GIFT toolbox. After clustering the obtained components, centrotype-based components were selected and considered as the stable decomposition result. The individual spatial patterns and time courses were obtained through a back reconstruction process.

### Functional Connectivity Analysis

**Figure 2:**
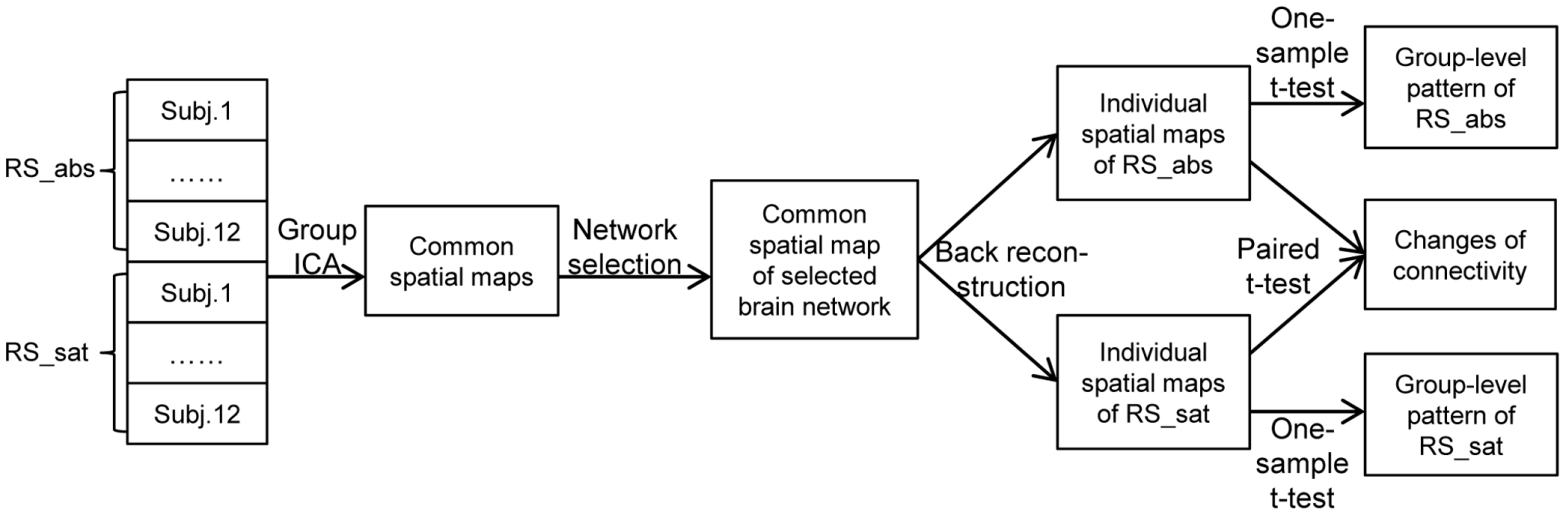
Flow diagram of functional connectivity analysis approach.

Brain networks of TNNs (i.e., aDMN and pDMN) and TPNs (i.e., ECN and SN) were visually selected from all subjects in both sessions. The individual spatial maps were normalized into *z*-score images, and were fed into the random-effect analysis using a one-sample *t*-test to infer the group-level activation pattern of each brain network in the two separate sessions. The differences in the functional connectivity of each network between the two sessions were explored using a paired *t*-test. A complete diagram of how functional connectivity was analyzed is illustrated in Fig. 2.

### Granger Causality Analysis (GCA)

Analysis of effective connectivity was facilitated by using GCA, which models one directional causality among multiple time series based on a VAR model [37]. The bivariate VAR model can be expressed mathematically as follows:
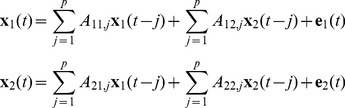
(1)


Here, **x**_1_(*t*) and **x**_2_(*t*) are the time series at time point *t*. Matrix *A* contains the regression coefficients, e.g., coefficient *A_12,j_* denotes the causal influence of **x**_2_(*t-j*) on the prediction of **x**_1_(*t*). **e**_1_(*t*) and **e**_2_(*t*) are the residual error at the time *t*. The model order *p* that represents the maximum time lag can be estimated using the Bayesian Information Criterion (BIC) [38]. When the residual error reaches the minimum, an *F*-test is used to estimate the statistical significance of the estimated model based on the residual error. A higher *F*-score means a stronger prediction of Granger causality between the two time series.

To investigate the effective connectivity among brain networks as well as among multiple brain regions, a MATLAB toolbox for GCA approach [39] was adopted to enable further analysis of the causal relations based on previous results from the functional connectivity analysis.

### Analysis of the Effective Connectivity among Brain Networks

For each individual in each session, the time courses corresponding to the brain networks (i.e., aDMN, pDMN, ECN, and SN) were obtained from the back-reconstruction process in group ICA. Then they were band-pass filtered (0.01–0.08 Hz), and normalized into zero mean and unit variance. The resulted time courses were concatenated across twenty-one subjects and served as the inputs for the GCA so as to reveal the causal interactions among brain networks in RS_abs and RS_sat.

### Definition of Regions of Interest (ROIs)

In previous studies of smoking addiction using resting-state fMRI [7], [12]–[15], brain regions such as the insula, para−/hippocampus, caudate, thalamus, pre−/cuneus, anterior cingulate cortex (ACC), posterior cingulate cortex, supplementary motor area (SMA), ventromedial prefrontal cortex (vmPFC, equivalent to Brodmann area 10) and dorsolateral prefrontal cortex (dlPFC, roughly equivalent to Brodmann areas 9 and 46) were found to be highly influenced by smoking, and can thus be treated as the critical smoking-related brain regions. In our framework, if the afore mentioned brain regions showed significant changes by previous paired *t*-test in the functional connectivity analysis, their peak *t*-value related voxels would be picked out. A selected peak voxel together with its surrounding 26 voxels (i.e., 27 voxels, 9×9×9 mm^3^) were defined as an ROI.

### Analysis of Effective Connectivity among ROIs

The average BOLD time series within each ROI was calculated from the fMRI data of each subject in each session, and shifted to zero-mean in order to facilitate the concatenation across subjects. The GCA was applied on the concatenated time series to explore the causal relations among smoking related brain regions in the two sessions separately. To describe the interactions between ROIs in the causal network, we additionally used the following described out-in degree metrics in traditional graph-theoretic analysis [37]: (1) Out-degree of a node is the number of causal out-flow connections from the node to any other nodes. (2) In-degree of a node is the number of causal in-flow connections to the node from any other nodes. (3) Out-In degree is then defined as the difference between out-degree and in-degree. A node with a high positive out-in degree is regarded as a causal source, while a node with a high negative out-in degree is considered as a causal target.

## Results

### Functional Connectivity Analysis

**Figure 3:**
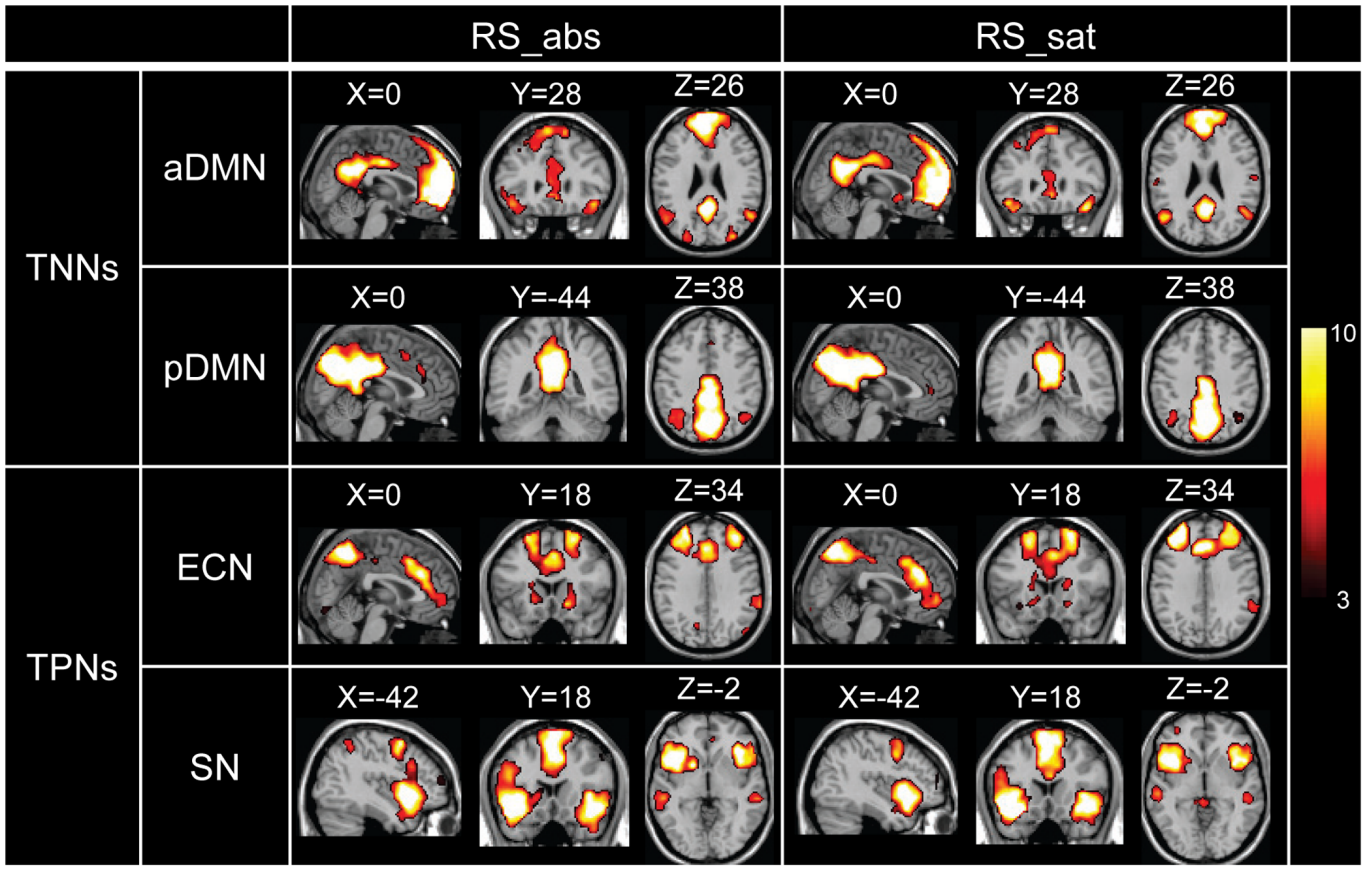
TNNs and TPNs via one-sample *t*-test of RS_abs and RS_sat separately (FDR corrected *p*<0.01).

The brain networks of TNNs (i.e., aDMN and pDMN) and TPNs (i.e., ECN and SN), indicated through one-sample *t*-test on back-reconstructed individual spatial maps, are shown in Fig. 3 (FDR corrected *p*<0.01). For TNNs, the aDMN mainly located in the ACC, and the medial and superior PFC; while the pDMN located in the PCC along with the precuneus and inferior parietal lobe. For TPNs, the ECN mainly contained parts of the anterior and medial PFC, as well as the superior parietal lobe; and the SN contained areas of the insula, ACC and anterior PFC.

The changes of functional connectivity revealed by paired *t*-test are listed in Table 2 (uncorrected *p*<0.005). In the resting-state of smoking abstinence, enhanced functional connectivity in both the aDMN and pDMN was indicated. In these two TNNs, hemodynamic responses were increased in the dorsal/rostral ACC (dACC/rACC), SMA and dlPFC, as well as the middle occipital gyrus and inferior frontal/temporal gyrus in the condition of smoking abstinence; while after replenishment, the hemodynamic responses were enhanced in regions of the precuneus and postcentral gyrus. For the two TPNs (i.e., ECN and SN), the parahippocampus, insula, as well as the inferior occipital gyrus and precentral gyrus showed the increased hemodynamic responses under the deprivation condition; whereas in smoking satiety, the hemodynamic responses were enhanced in regions such as the vmPFC.

**Table 2:** Changes of functional connectivity in each networks revealed by paired *t*-test at uncorrected *p*<0.005.

		Anatomical location	NmVx	Peak *t*-value	MNI coordinates
**aDMN**	RS_abs>RS_sat	L Inferior frontal gyrus	28	4.836	(−54, 27, −3)
		L Superior temporal gyrus	62	4.804	(−54, −33, 18)
		R Middle occipital gyrus	36	5.115	(45, −81, 15)
		R Middle cingulate cortex	28	4.344	(15, 21, 30)
		*L Anterior cingulate cortex(dACC)*	32	4.181	(−6, 21, 30)
		R Middle cingulate cortex	46	4.889	(3, −39, 48)
		*L Supplementary motor area*	34	4.161	(−3, 12, 69)
	RS_sat>RS_abs	L Postcentral gyrus	35	4.196	(−57, −15, 18)
		L Angular gyrus	43	5.116	(−42, −69, 45)
**pDMN**	RS_abs>RS_sat	L Cerebellum posterior lobe	37	4.225	(−27, −66, −45)
		L Cerebellum anterior lobe	22	4.208	(−27, −42, −33)
		L Inferior temporal gyrus	27	3.586	(−42, −15, −27)
		*R Anterior cingulate cortex(rACC)*	38	5.077	(6, 27, 15)
		*R Middle frontal gyrus (dlPFC)*	171	5.787	(42, 36, 42)
		L Middle frontal gyrus	27	5.643	(−27, −6, 57)
	RS_sat>RS_abs	R Vermis	57	5.089	(3, −57, −12)
		L Supramarginal gyrus	37	3.802	(−48, −45, 24)
		*L Precuneus*	20	3.432	(−12, −57, 42)
**ECN**	RS_abs>RS_sat	L Cerebellum posterior lobe	24	5.202	(−15, −90, −27)
		*R Parahippocampus*	44	4.521	(21, −15, −18)
		R Inferior occipital gyrus	25	4.614	(36, −78, −3)
		R Supramarginal gyrus	38	3.985	(66, −27, 39)
		L Superior frontal gyrus	56	3.880	(−9, 39, 45)
		R Precentral gyrus	40	4.175	(45, 0, 42)
	RS_sat>RS_abs	L Superior temporal gyrus	20	3.971	(−54, −24, 6)
		R Middle cingulate cortex	94	4.590	(3, 30, 30)
		R Middle frontal gyrus	25	4.146	(24, 36, 33)
		R Superior frontal gyrus	84	5.133	(27, 30, 54)
**SN**	RS_abs>RS_sat	R Middle temporal pole	31	4.855	(42, 15, −33)
		L Cerebellum posterior lobe	34	4.244	(−30, −78, −21)
		*R Insula*	37	5.018	(39, 9, 6)
		R Middle frontal gyrus	71	4.851	(30, 33, 45)
	RS_sat>RS_abs	*L Superior frontal gyrus (vmPFC)*	21	4.170	(−27, 51, 0)

The cluster information is illustrated using anatomical location, cluster size in number of voxels, peak *t*-value and its MNI coordinates. The eight regions marked in italics are our selected ROIs.

**Figure 4:**
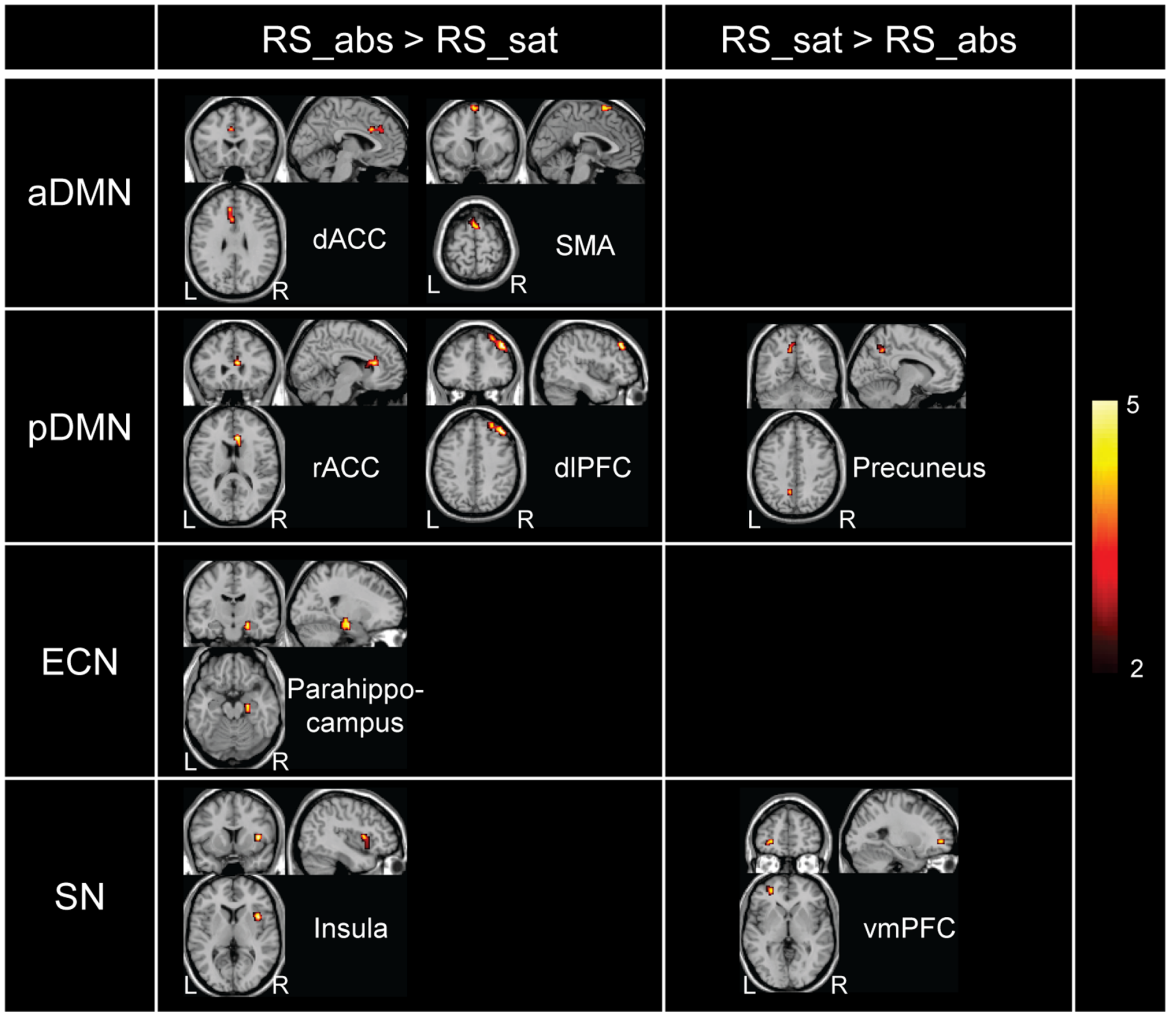
Eight ROIs located clusters with functional connectivity variation revealed using paired *t*-test (uncorrected *p*<0.005).

According to the ROI selection criteria described in previous section, we picked eight ROIs in total located in the following brain regions: the dACC, SMA, rACC, dlPFC, precuneus, parahippocampus, insula and vmPFC. These regions are displayed in Fig. 4 and marked with italic types in Table 2.

### Effective Connectivity Analysis

**Figure 5:**
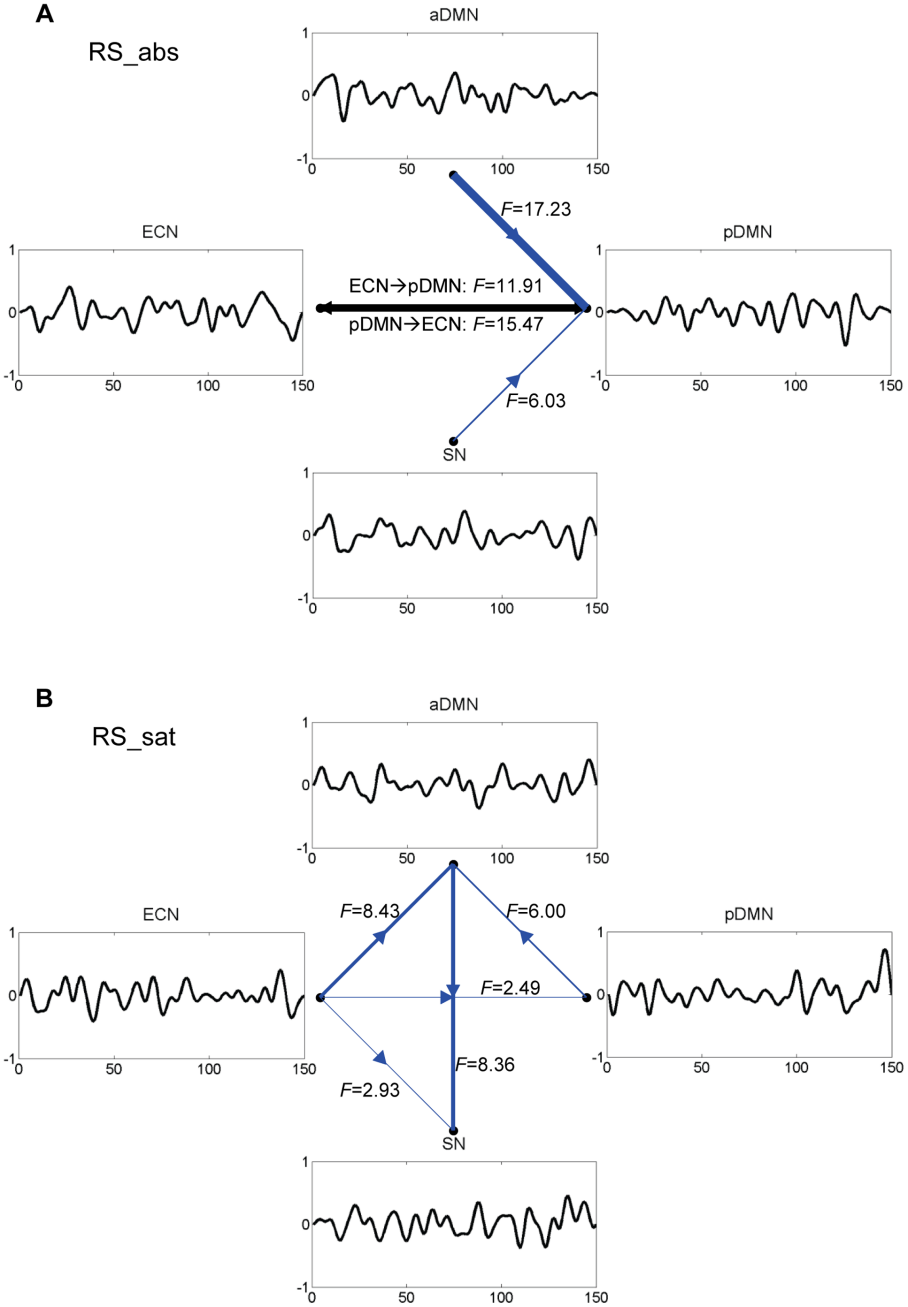
Causal interactions among brain networks. Causal interactions among aDMN, pDMN, ECN, and SN (FDR corrected *p*<0.01) in conditions of (A) smoking abstinence and (B) smoking satiety, together with the mean time course of each brain network. The reciprocal causal flows were revealed using black color, and the nonreciprocal ones were in blue color.

Fig. 5 shows the causal interactions among the brain networks of the aDMN, pDMN, ECN, and SN (FDR corrected *p*<0.01), together with the mean time course of each brain network. When smoking was replenished for the participants after abstinence, causal connection from SN to pDMN was reduced, whereas that from ECN/aDMN to SN was enhanced. Specifically, the ECN showed more active causal interactions with the other three brain networks in satiety condition. In these four causal nodes, the pDMN was a causal target in abstinence condition. After smoking replenishment, the SN turned to be a causal target, and the ECN became a causal source.

**Figure 6:**
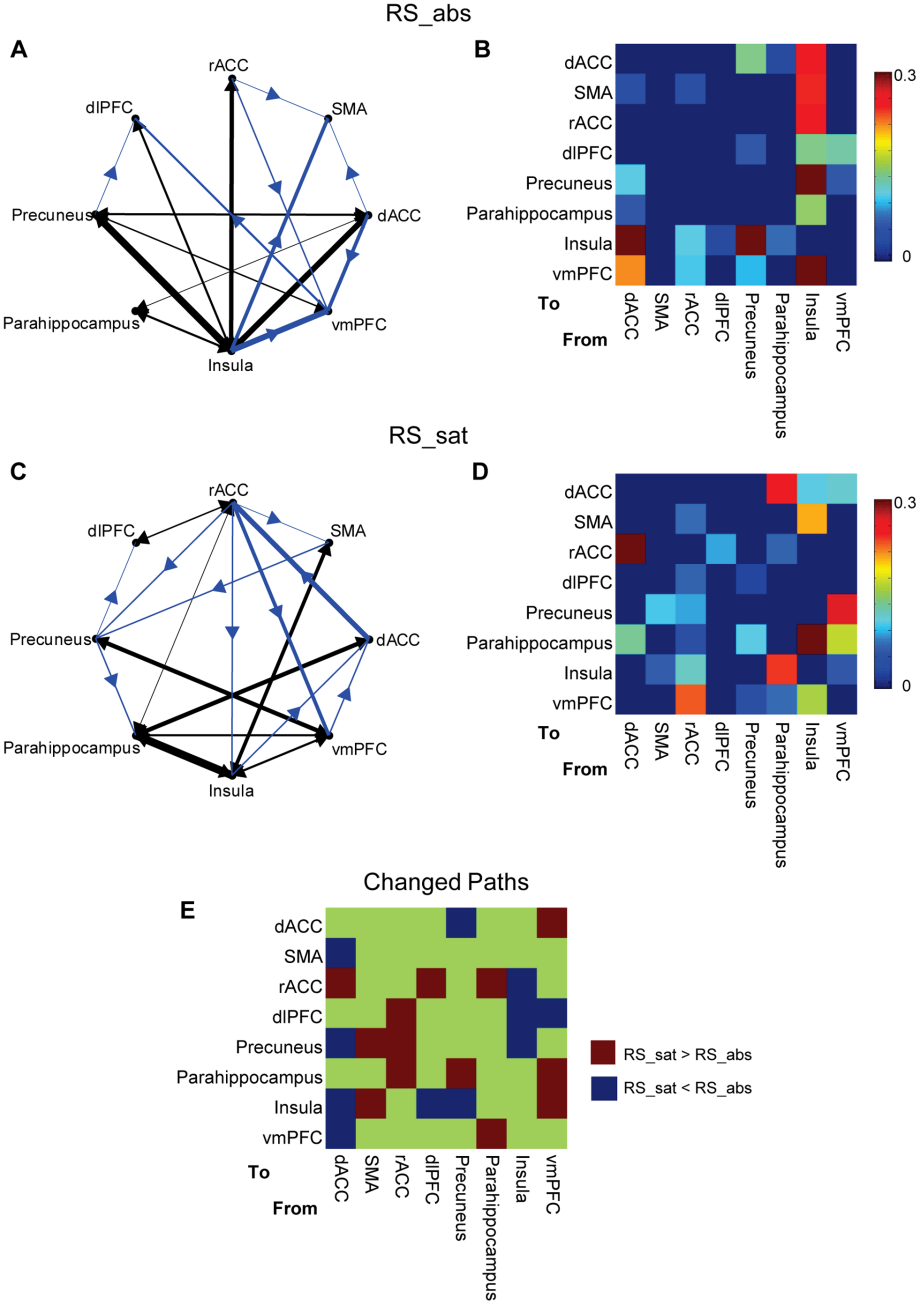
Causal interactions among selected ROIs. Causal interactions among selected eight ROIs in conditions of smoking abstinence and smoking satiety: (A) causal relationship of smoking abstinence at FDR corrected *p*<0.001, (B) significant path weight matrix of causal interactions in smoking abstinence; (C) causal relationship of smoking satiety at FDR corrected *p*<0.001, (D) significant path weight matrix of causal interactions in smoking satiety; (E) matrix indicating the changed causal paths in the satiety vs. abstinence condition. In (A) and (C), the reciprocal causal flows were revealed using black color, and the nonreciprocal ones were in blue color. In (B) and (D), the insignificant path weights are indicated in zero.

In Fig. 6, (A) and (C) display the causal relationships among eight ROIs before and after smoking (FDR corrected *p*<0.001); (B) and (D) are the corresponding significant path weight matrices. The causal degrees measured using *F*-score are listed in Table 3. For clear illustration, Fig. 6 (E) indicates the changed causal paths in the satiety vs. abstinence condition. Overall speaking, the insula and dACC had the increased inter-area causal connection in smoking abstinence, whereas the parahippocampus and rACC showed the enhanced causal interactions with the other ROIs after replenishment. Referring to the out-in degree metrics (Table 4), the SMA seemed to be a causal target in deprived state, and the rACC could be regarded as a causal source in satiety condition.

**Table 3:** The degree of the significant causal interactions in Fig. 6 (measured using *F*-score).

	From	To	*F*-score
**RS_abs**	dACC	SMA	3.61
		Precuneus	18.07
		Parahippocampus	7.18
		Insula	63.39
		vmPFC	36.24
	rACC	SMA	4.99
		Insula	19.64
		vmPFC	13.23
	dlPFC	Insula	7.59
	Precuneus	dACC	25.31
		dlPFC	7.30
		Insula	108.81
		vmPFC	15.90
	Parahippocampus	dACC	4.71
		Insula	15.00
	Insula	dACC	47.97
		SMA	43.31
		rACC	44.46
		dlPFC	22.61
		Precuneus	70.57
		Parahippocampus	24.92
		vmPFC	62.23
	vmPFC	dlPFC	19.66
		Precuneus	9.57
**RS_sat**	dACC	rACC	51.61
		Parahippocampus	12.65
	SMA	Precuneus	14.23
		Insula	4.73
	rACC	SMA	8.79
		dlPFC	7.96
		Precuneus	11.61
		Parahippocampus	3.45
		Insula	13.98
		vmPFC	33.10
	dlPFC	rACC	8.44
	Precuneus	dlPFC	4.29
		Parahippocampus	15.13
		vmPFC	4.78
	Parahippocampus	dACC	34.03
		rACC	11.10
		Insula	30.47
		vmPFC	3.25
	Insula	dACC	15.01
		SMA	31.32
		Parahippocampus	75.31
		vmPFC	23.08
	vmPFC	dACC	18.12
		Precuneus	42.16
		Parahippocampus	6.80
		Insula	8.92

**Table 4:** The out-in degree corresponding to the causal networks in Fig. 6.

	dACC	SMA	rACC	dlPFC	Precuneus	Parahippocampus	Insula	vmPFC
**RS_abs**	2	−3	2	−2	1	0	2	−2
**RS_sat**	−1	0	3	−1	0	−1	0	0

## Discussion

In this study, in order to investigate the neuronal mechanisms associated with smoking, we conducted a resting-state fMRI experiment in which twenty-one heavy smokers were scanned in two sessions: smoking abstinence followed by smoking satiety. We proposed a novel analysis framework combining ICA and GCA to inspect both functional and effective connectivity, mainly considering the brain networks of TNNs (i.e., aDMN and pDMN) and TPNs (i.e., ECN and SN). The causal interaction from SN to pDMN was reduced, and that from ECN/aDMN to SN was enhanced after smoking replenishment. The experimental results also showed that before and after smoking, both functional and effective connectivity changed in the brain regions of the insula, dACC, rACC, SMA, dlPFC, vmPFC, precuneus and parahippocampus. These findings did not only reflect the effect of smoking/nicotine on brain connectivity, but also could be explained by the presence and absence of withdrawal. Obviously, it demonstrated our hypothesis that the effect of smoking replenishment on deprived heavy smokers would influence both functional and effective connectivity.

Previous studies of smoking or nicotine addiction have mostly focused on functional connectivity in resting-state or task-related environments. Besides functional connectivity, we also paid attention to effective connectivity, which was represented by causal interactions. In our analysis framework, group ICA was applied to identify functionally connected brain networks. The time courses of specific functional networks were analyzed using GCA to reveal the causal relationship among these networks. Changes in functional connectivity were investigated using a paired *t*-test on back-reconstructed individual spatial maps and the results enabled selection of the ROIs for further GCA analysis to explore the causal interactions among crucial brain regions. Undeniably, our method was not the first one that combined ICA and GCA in fMRI analysis. However, different from the previous ICA-GCA approaches that measured the effective connectivity using only the network time courses derived from ICA [27]–[28] or only the BOLD time series of peak clusters located in ICA spatial maps [29]–[30], our framework seems more systematic and full-scaled as it concerns the effective connectivity with respect to both the large-scale brain networks and the brain regions of varied functional connectivity.

Granger causality analysis, which describes a statistical interpretation of causal interactions between sets of time series, was adopted to measure the effective connectivity in our experiment. Common criticisms regarding GCA for fMRI data are as follows [40]: (1) the spurious influence might be occurred because of the systematic differences across brain regions in hemodynamic delays; and (2) changes in directionality could be caused by the differences in hemodynamic coupling in different regions. However, it seems that the impact of variable hemodynamic delays is not severe when the sampling interval is as long as 2000 ms [41]. For the second criticism, we obtained the ROIs from a significant functional connectivity analysis using statistical testing rather than adopting the other atlas-based ROIs. The ROI definition rule was based on the hypothesis that the changes in functional connectivity would be caused together with the variations in effective connectivity, which was also demonstrated by our experiment results.

The four brain networks chosen in our study were aDMN, pDMN, ECN and SN. The first two are anterior and posterior parts of DMN. A previous study [7] reviewed the earlier research on resting-state functional connectivity in addiction, and proposed a hypothesized network model of nicotine addiction regarding these selected networks so that abstinence would result in one or more of the following observable changes: (1) enhanced connectivity within the DMN, (2) reduced connectivity within the ECN, (3) enhanced interactions between DMN and SN, and (4) reduced interactions between ECN and SN. Our functional connectivity results directly testified to the validity of the first hypothesis. The GCA result among brain networks could demonstrate the fourth hypothesis.

The ROIs revealed by the paired *t*-test were generally in agreement with the results of previous smoking research [7], [12]–[15]. Particularly, as a part of SN, the insula showed the enhanced hemodynamic responses and increased inter-area causal interactions in abstinent condition, giving the evidence of insula’s interoceptive effects in the addiction cycle that it tracks bodily changes and in turn interacts with other regions rather than acting solely [42]. An interesting finding is that the two ACC ROIs (i.e., dACC and rACC), which all showed the enhanced hemodynamic responses in deprived condition, performed differently in causal analysis. The dACC had the enhanced inter-area causal interactions during smoking abstinence, whereas the rACC showed the increased inter-area causal connection in satiety condition. A previous study [11] found that when taking them as seed-voxels, the dACC and rACC revealed the different resting-state whole-brain functional connectivity maps for healthy smokers, which potentially indicated that these two parts might also perform different roles in causal interactions. However, further studies are still needed to ascertain whether the sub-regions of the ACC has different mechanisms in smoking addiction.

Furthermore, neuropharmacological mechanisms could also relate the effect of smoking deprivation and replenishment on functional network activity. Nicotine acts on the nicotine acetylcholine receptors. In low concentrations, nicotine increases the activity of these receptors. It has been found that the nicotine acetylcholine receptors are associated with the dACC involved circuits [43], [44]. This may be a neural-level explanation of our experimental result that the dACC exhibited the enhanced hemodynamic responses and causal interactions in abstinent condition. Moreover, acetylcholine performs a role in toggling circuit dynamics between cortico-cortical feed-backward states (low acetylcholine levels) and thalamo-cortical feed-forward states (high acetylcholine levels) [45], which may reflect in parallel as the smoking induced suppression of DMN and enhancement of ECN activity at the system-level.

In our experiment, the scanning sessions were designed in the order of smoking abstinence followed by satiety, since our goal was to investigate the connectivity variations caused by smoking replenishment on the abstinent state. More specifically, we considered the abstinent state as a reference state of satiety, rather than treating these two states as two disconnected and separate conditions. However, it is possible that order effects such as more anxiety in the first session might have an impact on our findings [46]. A potential solution is to add one more resting-state session directly before the abstinence session so as to make the participants familiar with the scanning procedure. Moreover, since our experiment was based on measuring changes in blood flow, the elevation of blood pressure caused by smoking [47] might also confound the results. Exact impact of these factors are waiting for the future researches, and a variety of additional neuroscience measurements will be applied before a firm conclusion can be made about the effect of smoking on human brain functions.

### Conclusion

To investigate the brain connectivity associated with smoking addiction, we conducted a resting-state fMRI experiment and proposed an analysis framework combining ICA and GCA that can inspect both functional and effective connectivity in terms of brain networks and ROIs. The experimental results demonstrated our hypothesis that for deprived heavy smokers, smoking replenishment took effect on both functional connectivity and effective connectivity, involving brain networks such as TNNs and TPNs, as well as some crucial brain regions. Moreover, our proposed analysis framework could be applied across a range of neuroscience studies.
